# STUDY OF THE ANTEROLATERAL LIGAMENT OF THE KNEE IN FORMALIN-EMBEDDED CADAVERS

**DOI:** 10.1590/1413-785220172502162204

**Published:** 2017

**Authors:** Paloma Batista Almeida Fardin, Juliana Hott de Fúcio Lizardo, Josemberg da Silva Baptista

**Affiliations:** 1 Universidade Federal do Espírito Santo (UFES), Vitória, ES, Brazil.; 2 Universidade Federal do Espírito Santo, Department of Morphology, Laboratory of Applied Morphology (LEMA), Vitória, ES, Brazil.

**Keywords:** Knee, Ligaments, articular, Joint instability, Cadaver

## Abstract

**Objective:**

To verify the incidence and characterize morphologically the anterolateral ligament of the knee (ALL) in cadaveric samples of the collection of the Laboratory of Anatomy of the Department of Morphology of the Universidade Federal do Espírito Santo.

**Methods:**

Dissections and cross sections were performed for mesoscopic analysis of the anterolateral region of 15 knees preserved in 4% formalin solution in order to identify the ALL.

**Results:**

After dissection of the skin and subcutaneous tissue of the knee anterolateral region, it was possible to identify the iliotibial tract (ITT), the patellar ligament and the femoral biceps tendon. The ITT was removed from the Gerdy tubercle and the following structures were visualized: knee joint capsule, fibular collateral ligament and popliteal tendon. However, the ALL was not identified in any of the samples.

**Conclusions:**

The ALL could not be identified in any of the specimens studied, either through dissection or mesoscopic analysis. ***Level of Evidence III, Diagnosis Studies - Investigation of an Exam for Diagnosis.***

## RESUMO

**Objetivo:**

Verificar a incidência e possivelmente caracterizar morfologicamente o ligamento anterolateral do joelho (LAL) em amostras cadavéricas do acervo do Laboratório de Anatomia do Departamento de Morfologia da Universidade Federal do Espírito Santo.

**Métodos:**

Foram realizadas dissecações e secções transversais para análise mesoscópica da região anterolateral de 15 joelhos conservados em solução de formalina a 4% a fim de identificar o LAL.

**Resultados:**

Após a dissecação da pele e da tela subcutânea da região anterolateral dos joelhos foi possível identificar o trato iliotibial (TIT), o ligamento patelar e o tendão do músculo bíceps femoral. Após a desinserção do TIT no tubérculo de Gerdy as seguintes estruturas foram visualizadas: cápsula articular do joelho, o ligamento colateral fibular e o tendão do músculo poplíteo. Entretanto, o LAL não foi identificado em nenhuma das amostras.

**Conclusões:**

O LAL não pôde ser identificado em nenhum dos espécimes estudados, seja através da dissecação ou da análise mesoscópica. Nível de Evidência III, Estudos Diagnósticos - Investigação de um Exame para Diagnóstico.

## INTRODUCTION

In 1879, Segond^1^ described an avulsion fracture of the proximal anterolateral tibial region and mentioned the existence of a fibrous and resistant band that becomes taut by medial rotation. The term "Segond fracture" consequently describes an avulsion fracture of this region. After this description was made, some studies demonstrated the presence of a ligamentous structure between the lateral condyle of the femur and the anterolateral tibial region.^2^ ^-^ ^11^ In 2007, Vieira et al.^8^ introduced the term anterolateral ligament (ALL) of the knee to describe the ligament that originates in the lateral condyle of the femur anterior to the fibular collateral ligament (FCL) which has an oblique path in the anteroinferior direction with insertion in the proximal tibia between Gerdy's tubercle and the head of the fibula.

As a result of injury to the anterior cruciate ligament (ACL), many patients present anterolateral knee instability even after ligament reconstruction surgery.^12^ Considering that the ALL is not normally approched in reconstruction surgeries, it may be involved in destabilization of the knee after injury.^1^ ^,^ ^13^ This led to a broad scientific search to confirm and characterize the ALL.

Consequently, proper anatomical description of the ALL are extremely important in the clinical approach to knee ligament injuries, since the ALL seems to be involved in anterolateral stabilization and in limiting rotational movements of the knee.

Therefore, the objective of this study was to verify the incidence of the ALL in formalin-embedded cadavers, and after identification describe its characteristics and anatomical relationships.

## MATERIALS AND METHODS

This research was submitted to Plataforma Brasil and approved (Opinion 1,316,575). We studied 15 knees (8 right and 7 left) routinely fixed in 4% formalin solution to the human anatomy collection at the Department of Morphology at the Centro de Ciências em Saúde (CCS) at the Universidade Federal do Espírito Santo (UFES). With regard to the morphological characteristics of this collection, the average age in this sample was 50 years and the specimens were taken from male individuals. Only knees containing all cutaneous and fascial strata were included in the study; in other words, only those that had skin, subcutaneous tissues, and deep fasciae intact were selected. Specimens which had undergone any type of previous dissection, had malformations, scarring, or any sign of injury were not included in the sample.

**Figure 1:**
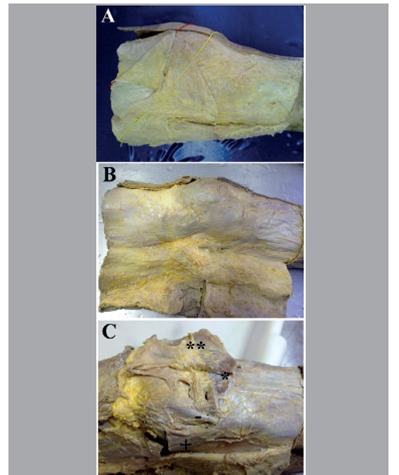
Photographs of the anterolateral region of the knees, right antimere. (A) Note the strata in the anterolateral region of the knee: skin folded back to the side, subcutaneous tissue folded back and marked by the yellow line, the iliotibial/fascia lata tract, partially folded back and marked by the red line. (B) Note that the skin and the subcutaneous tissue were folded back to demonstrate the iliotibial/fascia lata tract, patella, patellar ligament, and lateral retinaculum and crural fascia; (C) dissection of the iliotibial tract and the knee joint capsule: (*) detached Gerdy's tubercle; (**) iliotibial tract and articular capsule of the knee, folded back; (-) fibular collateral ligament; (+) tendon of the biceps femoris muscle.

**Figure 2:**
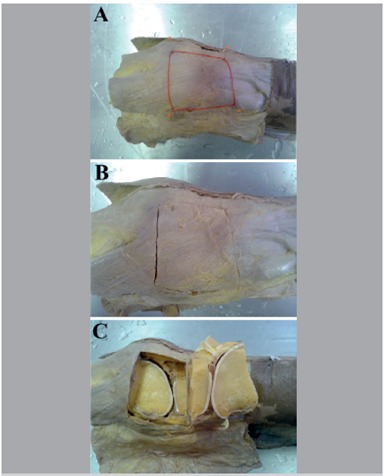
Photographs of the anterolateral region of the knees, right antimere. (A) Demarcation of the area of the block to be removed; (B) Sectioning of the block; (C) Careful removal of the block for mesoscopic study.

Dissection was performed carefully from the anatomical position and followed the methodology of Claes et al.^10^ First, skin and subcutaneous tissue were dissected from the medial to the lateral region of the knee in order to identify the iliotibial tract (ITT), the patellar ligament, and the tendon of the biceps femoris muscle (Figures 1A and B). The ITT was sectioned 5 cm from the lateral epicondyle of the femur and detached from Gerdy's tubercle in order to permit identification of the joint capsule, the FCL, and the popliteus muscle tendon. (Figure 1C) At this point of the dissection we expected to be able to identify the ALL, but this structure was not seen in the first 3 samples, contrary to the results found in the literature.^10^ ^,^ ^14^ With this in mind, the remain specimens (n=12) were dissected to expose the ITT, the patellar ligament, and the tendon of the biceps femoris muscle (Figures 2A and B). Next, an anatomical block of the anterolateral region of the knee was extracted via cross sections of approximately 7 cm at the level of Gerdy's tubercle and the lateral epicondyle of the femur, and sagittal sections of approximately

7 cm at the level of the lateral edge of the patella and the posterior edge of the femoral condyle and the fibula head (Figure 2C). This block was carefully removed and underwent micro-dissection and analysis in a stereomicroscope (Stemi2000C and AxioVision image analysis software, Zeiss, Germany - Laboratory of Applied Morpology - LEMA). A cross section was performed along the articular line of the knee to permit micro-dissection, identification, and isolation of the anterolateral structures such as the synovial membrane, the joint capsule, and the fibrous bands which form the ligaments in the region in order to identify the ALL.

## RESULTS

**Figure 3:**
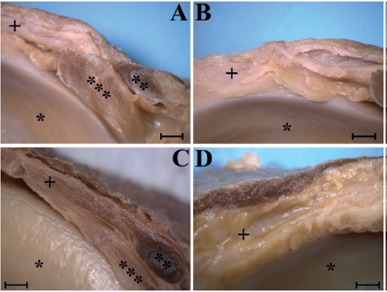
Mesoscopic-level photographs of the anterolateral region of 4 knees, right antimere (A-D). Scale bar 2mm. (*) Lateral meniscus, (**) fibular collateral ligament, (***) popliteus muscle tendon. (+) Indicates the presumed region of the anterolateral ligament of the knee, namely the region between the joint capsule and the lateral retinaculum. Note that there is no structure compatible with a ligament, although this region comparatively presents a denser constitution in A and C, and is more lax in B and D. Medial to the region indicated by the (+) is the synovial membrane, and laterally the iliotibial tract.

This methodology allowed easy identification of the skin, subcutaneous tissue, ITT, patellar ligament, tendon of the biceps femoris muscle, FCL and its relation to the tendon of the popliteus muscle, the joint capsule, and the lateral meniscus (Figures 3A-D); however, in none of the samples we were able to isolate the ALL as a distinct fibrous band.

## DISCUSSION

Although the first description of the ALL of the knee was made by Segond^1^ in 1879, recent studies have given to this structure international prominence in the field of anatomy and surgery. In this context, it is important to highlight that all these investigations used (in whole or in part) samples from fresh cadavers^10^ ^,^ ^14^ ^-^ ^16^ or studied living individuals through images such as magnetic resonance.^15^ ^,^ ^17^ ^,^ ^18^ However, there is no consensus among the results of these studies on the anatomical characteristics of the ALL:^10^ ^,^ ^16^ ^,^ ^19^ ^,^ ^20^ while some studies verified the presence of the ALL in most of the samples,^10^ ^,^ ^15^ ^,^ ^16^ others did not identify this ligament in even half of the specimens,^21^ ^,^ ^22^ and one study show no ALL in their samples.^19^ Furthermore, contradictory results exist within the same investigation: Vincent et al.^9^ identified the ALL of the knee as a capsular structure in individuals during arthroscopic knee surgery, while in its cadaver sample this ligament was found to be intracapsular. Moreover, there is no consensus about the precise location, form, and fixation of the ALL, unlike other ligaments in this region.

We were motivated by these contradictory results, and initially we adopted the dissection method which was used in other studies.^10^ ^,^ ^23^ Even though the same method of Claes et al.^10^ and Daggett et al.^23^ was adopted, we were not able to identify the ALL in any of our specimens, in contrast with the findings by these authors. In the latter study, the authors dissected and carefully folded back the ITT in order to avoid interference with the attachment of the ALL, since this ligament is presumably adhered to the ITT. Next, Daggett et al.^23^ were able to identify the ALL as originating in the lateral epicondyle of the femur and inserting between Gerdy's tubercle and the fibular head. However, these authors obtained better visualization of this anatomy after sectioning the knee capsule, where it was seen that the ligament originates posterior to the lateral epicondyle and also inserts into the lateral meniscus, in addition to the insertions which have already been described. Again, although we used the same careful dissection technique, it was not possible to verify the ALL. It should be noted, however, that when analyzing the images obtained by Claes et al.^10^ and Daggett et al.^23^ using similar dissection techniques, the structures referred to as the ALL of the knee are not identical: while Claes et al.^10^ showed a chordoid and cylindrical structure, Daggett et al.^23^ showed the ALL flat or laminar with less precise form. This may suggest an artifact of the dissection with consequent bilamination of the ITT, since the authors themselves stated that the ITT should be carefully dissected considering that the ALL is closely inserted into its deep surface.^23^

The study conducted by Shea et al.^20^ ^)^ also featured contradictions between the results and the discussion; these authors identified the ALL in only 1 of the 8 specimens studied (specimen age: 3 and 4 months, 1, 2, 3, 8, and 10 years), even though they used the same dissection process which was applied in this study and by the other authors.^10^ ^,^ ^23^ As an argument, Shea et al.^20^ stated that this result arises from the possibility that the ALL is a ligament which develops during growth and therefore would only be present in older specimens, which would explain the absence of the ligament in the samples studied. However, their data show that the one specimen in which it was possible to identify the ALL was only 1 year of age.

The hypothesis that the ALL might actually be a thickening of the joint capsule and not necessarily a ligament^24^ would explain the discrepant results derived from studies involving this ligament. In fact, the data obtained so far are not sufficient to morphologically characterize the ALL as a ligament. Corroborating this idea as well as our results, Capo et al.^19^ was unable to identify the ALL in any of the cadaver samples they studied using ultrasound. What these authors observed was a structure located in the anterolateral region of the knee that was suggested as a thickening of the ITT or even a fascia, and although the specimens were subsequently dissected, the absence of the ALL was confirmed.

Considering that dissection did not allow us to identify the ALL in the present study, we used mesoscopy, a methodology which has not yet been explored in the literature. It is important to highlight that micro-dissection using mesoscopy, after creating a transverse section along the joint, presented the intact strata of the anterolateral region of the knee, completely eliminating the bias of bilaminating the fascial strata or the ITT at the time of dissection. Again, according to the results obtained by the dissection the ALL was not identified. One limitation of our study may be the use of a restricted sample compared with previous studies,^10^ ^,^ ^15^ ^,^ ^16^ although several current studies have used fewer specimens.^11^ ^,^ ^19^ ^,^ ^20^ ^,^ ^23^

The methodology applied in this present study allowed us to assert that it was not possible to identify the ALL of the knee as an isolated structure with specific anatomical features (like other ligaments in the region), as has been indicated in research in this area.^10^ ^,^ ^14^ ^,^ ^15^ Therefore, the results we obtained along with the methodology we employed characterize the original nature of our research.

In conclusion, we reaffirm that the primary and possible explanation for our conflicting results are the artifact of dissection with consequent bilamination of the structures of the anterolateral region: namely the ITT, the lateral retinaculum of the knee, or the knee joint capsule. Consequently, the structure known as the ALL would not necessarily be an individualized ligament but rather part of another structure of the knee which in the cross section does not resemble the anatomical characteristics of the other ligaments of the region.

## CONCLUSION

It was no possible to indentify the ALL of the knee as an individualized ligament in formalin-embedded cadavers. This result does not conclude the discussion, but suggests the need for future research using larger samples that effectively represent a population.
